# Iodine supplementation for pregnancy in Portugal: identification of nutritional composition of iodine containing supplements and characterization of pharmacy dispenses from 2008-2022

**DOI:** 10.3389/fendo.2025.1582610

**Published:** 2025-05-22

**Authors:** Sarai Isabel Machado, Susana Roque, Maria Lopes-Pereira, Maria José Costeira, Patrício Costa, Nuno Borges, Ruben Pereira, Zilda Mendes, António Teixeira Rodrigues, Joana Almeida Palha

**Affiliations:** ^1^ Life and Health Sciences Research Institute (ICVS), School of Medicine, University of Minho, Braga, Portugal; ^2^ Hospital de Braga, Braga, Portugal; ^3^ Hospital da Senhora da Oliveira Guimarães, Guimarães, Portugal; ^4^ Faculty of Nutrition and Food Sciences, University of Porto, Porto, Portugal; ^5^ Centre for Health Evaluation & Research of the National Association of Pharmacies (CEFAR-ANF), Lisboa, Portugal

**Keywords:** pregnancy, public health, iodine, iodine deficiency, supplementation

## Abstract

**Background:**

Iodine is essential for the proper development of the central nervous system. In Portugal, despite of self-reports of iodine supplements intake by pregnant women, iodine deficiency still prevails. This study intended to characterize the pharmacy sales of iodine-containing supplements and their use by women in Portugal.

**Methods:**

Data from all available supplements with indication for use in the prenatal time, for the period 2008-2022, was obtained from the Portuguese Association of Pharmacies. To infer from the use of iodine supplements by women during preconception, pregnancy and lactation, pharmacy dispenses, between 2019 and 2021, were collected from a sample of female consumers, identified with at least one prescription of iodine-containing supplements.

**Results:**

Eighty-eight per cent of the iodine-containing supplements state the recommended iodine concentration. The annual sales increased continuously, reaching an estimated of 109 µg per day in 2022. Estimated mean duration of supplementation to women prescribed with iodine-containing supplements for preconception/pregnancy/lactation was of 4.5 months (SD = 3.8) and less than 1% of women was covered for the advisable period of 18 months.

**Conclusion:**

The estimated duration of iodine supplementation to women during preconception, pregnancy and lactation is below recommendations. Additional public health measures, such as universal salt iodization and literacy campaigns are needed to ensure iodine sufficiency to women and their developing fetus.

## Introduction

Pregnancy represents a particular time of susceptibility to malnutrition due to increased demands of nutrients (1–3). Therefore, current international recommendations advise the supplementation of folic acid, iron and iodine. Legislation and usage of micronutrient supplementation varies among countries and should be adjusted to target and prevent the risk of deficiencies in each population group (4, 5).

Portuguese women of childbearing age and pregnant were identified as iodine deficient in 2009 (6, 7). This led the health authorities, in 2013, to recommend daily iodine supplementation of 150-200 µg, in the form of potassium iodide during conception, pregnancy and lactation (8). Despite of over 70% of women in the Azores Islands, Covilhã and Minho region and 56% in Porto, reporting compliance with the supplement recommendation, pregnant women are still iodine deficient (median urinary iodine concentration below 150 µg/L) (9–12). These findings are concerning, given the need of iodine for the synthesis of thyroid hormones, which are essential for the proper development of the central nervous system (13) and for children to achieve their full intellectual performance (14). Iodine is mainly obtained through diet (mostly dairy products and seafood) (15) and iodine content in food depends on the geographic locations and production/agricultural practices (16). To maintain adequate intake and prevent iodine deficiency, many countries adopted food fortification strategies, particularly through iodized salt, currently covering 89% of households worldwide (17, 18). In Portugal, iodized salt is mandatory in the school age children’s canteens, which seems not to have been adopted (19). As for the general population, iodized salt is voluntary and represent a minor contributor to iodine intake, corresponding to only 11% of total salt sales (20). Irrespectively of the policy on iodized salt, iodine supplementation is recommended to pregnant women, given the higher requirements of iodine as the production of thyroid hormones increases (17).

The evidence on the lack of efficacy of supplementation measures in ensuring adequate iodine intake in Portuguese pregnant women raised questions about compliance and on whether timing of initiation is sufficient to recover iodine storages and promote an adequate status during pregnancy. To obtain evidence for whether additional health policy recommendations are needed, the present study characterizes the composition, availability, and yearly sales (2008 to 2022) of iodine-containing supplements in Portuguese pharmacies. In addition, from medically prescribed supplement dispenses to women, a cohort of female was identified to estimate the duration of iodine-containing supplements intake.

## Methods

### Characterization of iodine-containing supplements composition, availability, and evolution of sales from 2008 to 2022

Data from iodine-containing supplements sold in Portuguese pharmacies from 2008 to 2022 was provided by the Centre for Health Evaluation and Research, National Association of Pharmacies, that encompasses 94% of the Portuguese pharmacies. Supplements were classified as “medicinal supplements” (can only be sold in pharmacies, some of which requiring medical prescription, and are regulated by the National Authority of Medicines and Health Products) or as “food supplements” (can also be sold in the mass market and are regulated by the General Directorate for Food and Veterinary). All iodine-containing supplements with indication for use during the prenatal time, except those in liquid presentation (such as enteral feeding formulas as they usually target malnutrition), were considered. Data was retrieved from 2008 to 2022 sales on community pharmacies (iodine containing “medicinal” and “food” supplements) and mass market (for the 15 most sold iodine-containing “food supplements”). The share of mass market sales was provided by the company Health Market Research (it contains data mostly from onsite sales) (the information retrieved does not distinguish online from onsite sales).

Information on the products (composition on iodine, iron, folic acid and selenium; posology and dosage form) was obtained from the summary of product characteristics and, whenever necessary, complemented through the human medicinal products database (National Authority of Medicines and Health Products, I.P.) and web search.

Total iodine sold was calculated using iodine-containing supplement sales multiplied by the number of dosages per unit sold and the respective iodine content. Assuming that all iodine supplements sold are intended for women in preconception, pregnant, and/or lactating, an estimation was made for iodine sold per birth, per day. The number of births each year was retrieved from the National Institute of Statistics.

### Iodine compliance assessment from anonymized individual-level dispenses data of iodine-containing supplements

Given that iodine supplementation is recommended for preconception, pregnancy, and lactation, we considered that aggregated data from pharmacy dispenses could help clarify for how long the supplements were being taken. For that, anonymized data on pharmacy dispenses of iodine-containing supplements, obtained between 2018 and 2022, associated to consumers with at least one prescription of iodine-containing supplements was collected.

#### Inclusion criteria

Female consumers with dispenses of iodine-containing supplements with at least one prescription, between 2019 and 2021.

#### Exclusion criteria

Pharmacy dispenses of consumers with undetermined gender or males. Consumers with iodine-containing supplement dispenses in 2018 and/or 2022 (due to the possibility of underestimating the duration of supplementation, as they could be taking supplements in 2017 and 2023, for which we did not have data). Consumer identifiers with more than one attributed tax identification number to prescriptions (without univocal relation, and therefore not possible to distinguish individual consumers). Consumers younger than 18 or older than 45 years of age (considered outside the childbearing age). Consumers with dispenses of iodine supplements for periods longer than 18 months (since these could be taken iodine supplements for reasons other than pregnancy). Consumers with time gaps between dispenses above 18 months (since these could constitute more than one pregnancy).

**Figure 1:**
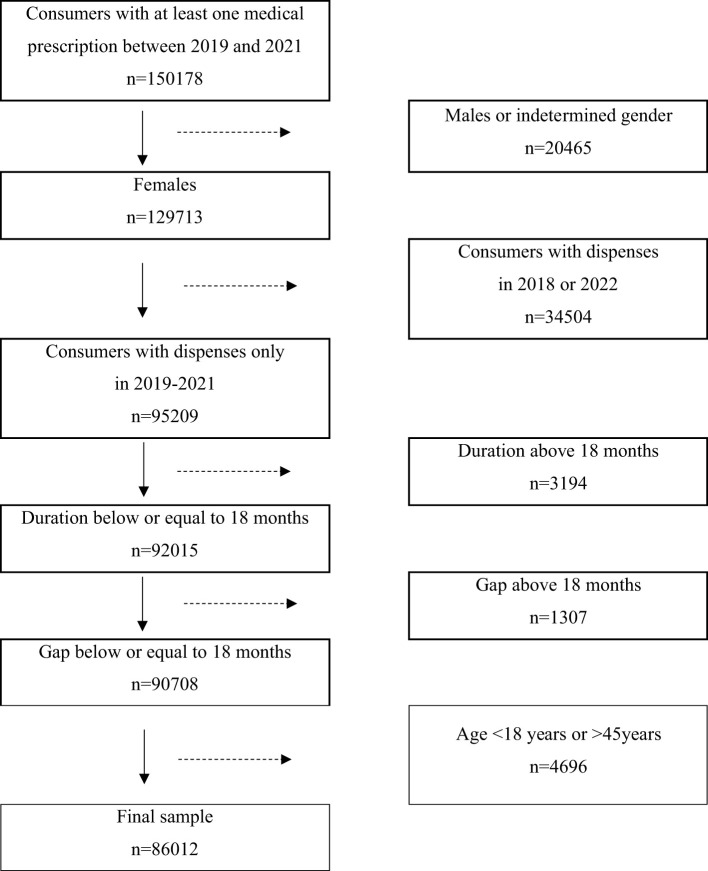
Flowchart of the study cohort.

Upon application of the exclusion criteria to the initial 150178 consumers, the final sample comprised 86012 women (**Figure 1**). From each woman retrieved through a medical prescription, all other iodine-containing supplement sales were also obtained, irrespectively of whether associated with another prescription or not. Iodine availability and estimated supplementation coverage in months were assessed by the sum of units dispensed, iodine content of the supplement purchased, and dosage form.

### Statistical analysis

The descriptive analysis of supplements was performed using Microsoft^®^ Excel for Mac, Version 16.83, and statistical analysis was conducted using IBM SPSS, 29.0 for Mac. Data was analyzed for normality using the Shapiro-Wilk test, skewness, kurtosis coefficients, and histogram. An interrupted time series analysis, presented as a segmented linear regression, was conducted to assess iodine supplement sales before and after 2013, the year in which the recommendation for iodine supplementation to women during preconception, pregnancy and lactation, was issued in Portugal (21, 22). Statistical significance was considered for a p-value below 0.05.

## Results

### Characterization of iodine-containing supplements composition, availability and sales

**Figure 2:**
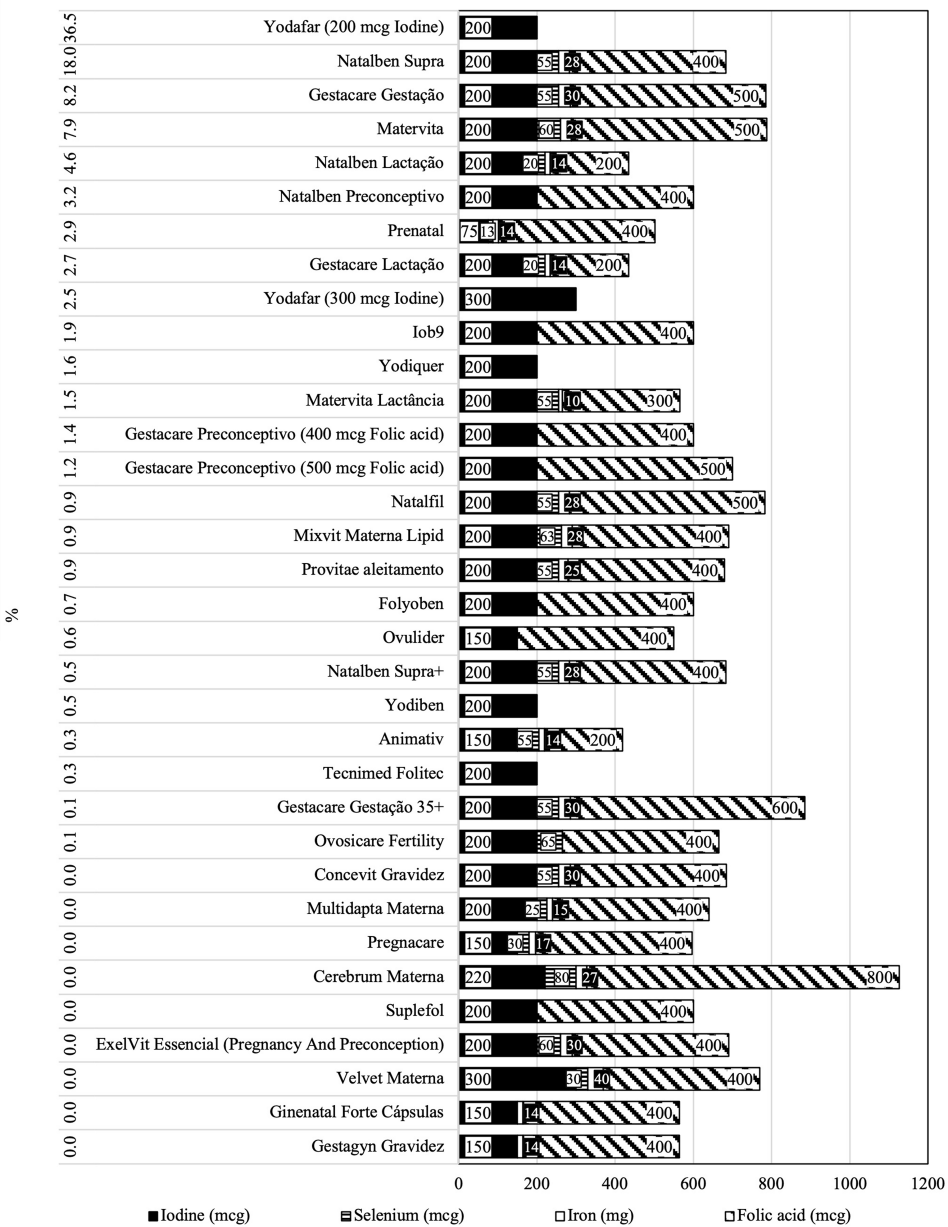
Nutritional composition in iodine, folic acid, iron and selenium, sorted by best-selling iodine -containing supplements in percentage sales.

From 2008 to 2022 sales, 34 iodine-containing supplement presentations were identified, 28 of which were classified as “food supplements” (27 multiple micronutrient formulation and 1 isolated iodine formulation) and 6 as “medicinal supplements” (3 multiple micronutrient formulation and 3 isolated iodine formulation). The nutritional composition of all iodine-containing supplements is presented in **Figure 2**, sorted by the percentage of total sales. Eighty eight percent of all supplements presented iodine content within the recommendations (150 - 200 µg). Three supplements had iodine content above recommendations (220, 300, and 300 µg, two classified as “food supplements” and one as “medicinal supplement”) and one had iodine content below the recommendations (75 µg, classified as “medicinal supplement”). Of the 30 multiple micronutrients supplements, 97% also had folic acid, 73% iron and 70% selenium in their composition. The 15 most sold iodine-containing “food supplements” represented 78% of iodine-containing food supplements sales in pharmacies. Of these 15 iodine-containing “food supplements”, 89% of sales were attributed to pharmacies and 11% to mass market.

**Figure 3:**
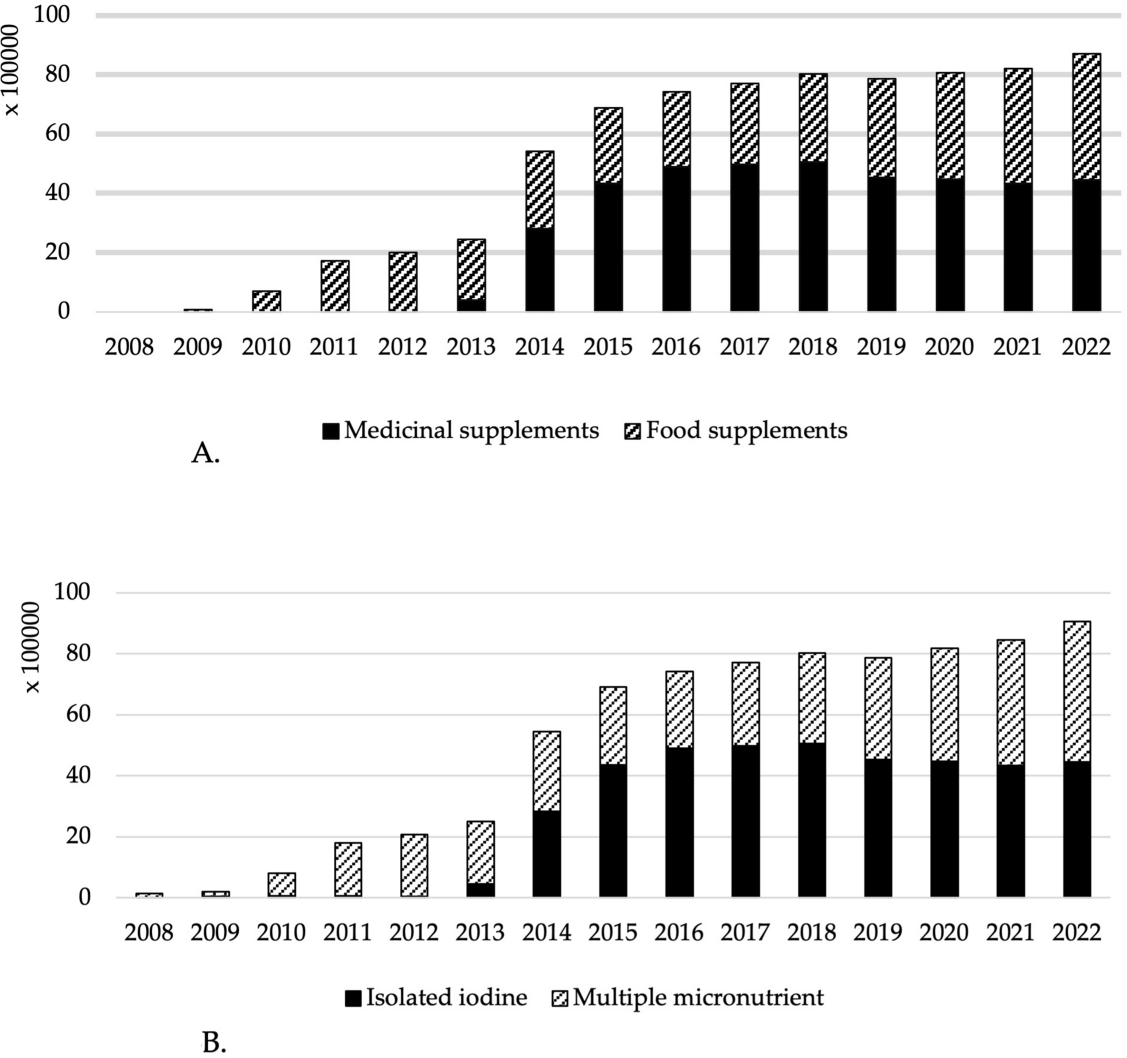
**(A)** Iodine sold (µg) per day attributed to medicinal supplements and food supplements from 2008 to 2022. **(B)** Iodine sold (µg) per day attributed to multiple micronutrient or isolated iodine formulas from 2008 to 2022.

The sales were next stratified based on formulation and requirement of prescription. While before the recommendation (2013) of the health authorities on the supplementation to women during preconception, pregnancy and lactation, isolated iodine represented a minute fraction of the iodine sales, it rapidly increased thereafter, reaching 49% of total sales in 2022 (**Figure 3A**). Of notice, before the recommendation in 2013, most sales were from “food supplements”, and “medicinal supplements” sales greatly increased thereafter, reaching 53% of total iodine-containing supplement sales in 2022 (**Figure 3B**).

Unitary sales, iodine sold, and year-over-year growth are presented in **Table 1**. The highest year-over-year unitary iodine-containing supplement sales growth was observed in 2010 and 2014 (158% and 90%, respectively). Iodine sold per day covers an estimated 56% of the recommendation requirements (114 vs. 200 µg/day) for the births of 2022 (**Table 1**).

**Table 1:** Iodine-containing supplements sales from 2008 to 2022.

Year	Units sold	Units sold growth	Number of products	Presentations growth	Number of births		Iodine sold per day per birth (μg)	*Including the estimation of sales from pharmacies and mass market (μg)
2008	22782		1		103541	1		–
2009	22455	-1%	3	200%	98430	2		–
2010	57906	158%	6	100%	100280	8		–
2011	104733	81%	9	50%	95823	19		–
2012	108024	3%	9	0%	88969	23		–
2013	122267	13%	15	67%	82064	30		–
2014	232421	90%	18	20%	81591	67		–
2015	282749	22%	22	22%	84584	82		–
2016	303835	7%	24	9%	86281	86		–
2017	322214	6%	26	8%	85315	90		–
2018	339792	5%	25	-4%	86256	93		94
2019	333195	-2%	24	-4%	85963	92		101
2020	367202	10%	29	21%	83907	98		104
2021	389944	6%	30	3%	78890	107		117
2022	423044	8%	28	-7%	82987	109		114

*Iodine sold per day per birth including the estimation of sales from pharmacies and mass market.

**Figure 4:**
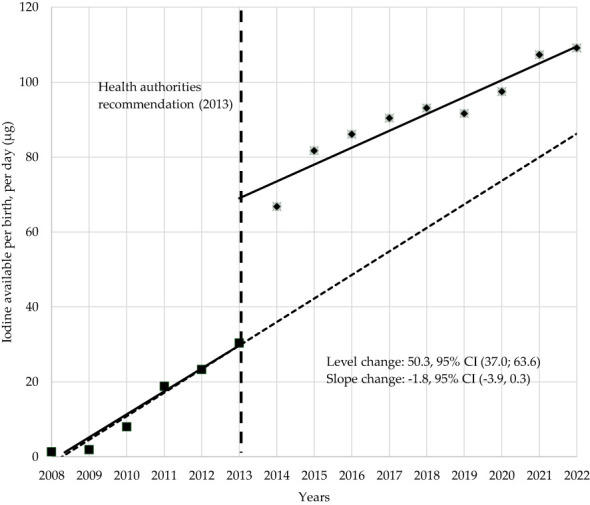
Segmented regression of iodine available, per birth, per day, before and after 2013.

To understand the influence of the health authorities’ recommendations on iodine supplementation, a separate analysis was made, before and after 2013 (**Figure 4**). In the year of the recommendation, 2013, a significant estimated 50.3 µg/day per birth increase was observed (p < 0.001, CI 95% = [37.0; 63.6]. No differences were found regarding the growth trend before and after the 2013 (p = 0.088, CI 95% = [-3.9; 0.3]). The growth trend from 2008 to 2022 indicates that iodine available increased each year an estimated 6.3 µg/day per birth (p < 0.001, CI 95% = [4.4; 8.1]).

### Duration of iodine-containing supplements intake

From the sample of 86012 women (**Table 2**), between 2019 and 2021, the median duration of supplementation was of 4.5 months (IQR = 5.0). Eighty three percent of women dispenses did not cover the expected duration of pregnancy (9 months), and less than 1% had iodine available for the recommended period of 18 months (3 months before conception, 9 months of pregnancy, and 6 months during lactation) (5).

**Table 2:** Sample characteristics and iodine supplementation coverage.

Sample characterization (n=86012)	M (SD)
Age (years)	32 (6)
Number of dispenses	2 (1)
Iodine content per dose (µg)	206 (5)
Supplementation coverage (months)	% women
0-1	<1%
1-2	40%
2-3	5%
3-4	15%
4-5	2%
5-6	10%
6-7	10%
7-8	1%
8-9	6%
9-10	1%
10-11	3%
11-12	2%
12-13	<1%
13-14	1%
14-15	<1%
15-16	3%
16-17	2%
17-18	<1%
Grand Total	100%

## Discussion

This study shows that availability of iodine-containing supplements in pharmacies increased throughout the years, particularly after the recommendation by the national health authorities, in 2013, for iodine supplementation to women during preconception, pregnancy, and lactation. Most presentations have iodine content within the recommended content per serve (150 - 200 µg). Despite of a great variety of presentations categorized as “food supplements”, most iodine sold is in the format of “medicinal supplements” and require medical prescription. Interestingly, with respect to formulation, the multiple micronutrient supplements have now a similar contribution to iodine sold as isolated iodine supplements (51% and 49%, respectively). This is in accordance with information reported by Portuguese pregnant women (9). Of notice, most multiple micronutrients supplements also contained folic acid, iron and selenium, elements well described to be relevant during pregnancy. In Portugal, there seems to be adequacy in folic acid (supplementation is well implemented), and in selenium (23), while iron deficiency is still prevalent (24). To highlight that both selenium and iron availability are known to affect thyroid function and modulate the response to iodine prophylaxis (25).

When considering the overall annual sales, and the number of births, the total amount of iodine sold from supplements per day, per birth, reached a maximum of 114 µg/day in 2022, which is quite below the proposed for supplementation (200 µg/day). The contribution of iodine from supplements, together with dietary intake, should reach 250 µg/day during pregnancy and lactation, as recommended by the World Health Organization United Nations Children’s Fund-UNICEF) and the Iodine Global Network (26). Globally, there was an increase in iodine-containing supplement sales and estimated iodine sold per birth, since 2008, with two major highs in 2010 (the year after the first studies reporting iodine deficiency in pregnant Portuguese women) and 2014 (the year after the recommendation on iodine supplementation by the national health authorities). Supplement sales have now stabilized.

Analysis from the cohort obtained from pharmacy dispenses show that compliance with the recommendation is partial; less than 1% of women take iodine-supplements during the suggested 18 months, with a median of 4.5 months of duration (5, 8). Women are therefore likely to initiate supplementation when already pregnant and/or interrupt supplementation before the end of lactation. This is in accordance with the data presented for the Porto region of Portugal, where only 56% of women reporting the use of iodine-containing supplements initiated intake earlier than the sixth week of pregnancy (11). The data also supports the hypothesis that women may terminate iodine supplement intake by the end of the first trimester, which is the time when folic acid intake is generally completed (27). Of notice, a shorter than expected period of iodine-supplement may justify that the urinary iodine levels remain low in Portuguese pregnant women, despite of the reported over 70% compliance with iodine supplementation (9, 10, 12). Irrespectively of the reason, women are not being adequately informed on and/or to the adequate timings for iodine supplementation. This calls for the need of literacy campaigns to health professionals (28) and to women (29). The observation that pharmacy sales cover around 89% of iodine prenatal food supplement sales in the country creates an opportunity to promote literacy campaigns in that setting.

The study presents some limitations. When overall sales alone are considered, the total iodine intake value can be overestimated, since men and non-pregnant women were not excluded (even though analysis focused on supplements targeting women during preconception, pregnancy and lactation). When the data is considered based on the dispenses, actual intake and compliance could have been overestimated since women may purchase iodine supplements without fiscal number or individual healthcare identifier. Sales of iodine-containing supplements do not assure that women will take them; in such circumstance the data presented may be overestimated. No additional information, such as on health status, living conditions, or preconception/pregnancy/lactating status, which could influence the purchasing behavior.

## Conclusion

This study contributes to the evidence that iodine intake by women during preconception, pregnancy and lactation is not achieving the levels considered sufficient. Altogether, the current evidence in Portugal supports the national health authorities to implement additional measures to ensure adequate iodine intake to women who intend to become pregnant, pregnant and through lactation. These include universal salt iodization and literacy campaigns to women, health professionals and the public.

## Data Availability

The data analyzed in this study is subject to the following licenses/restrictions: The data generated in the current study will be available from the authors on reasonable request. Requests to access these datasets should be directed to JP, japalha@med.uminho.pt.
